# Identification of the Adenovirus E4orf4 Protein Binding Site on the B55α and Cdc55 Regulatory Subunits of PP2A: Implications for PP2A Function, Tumor Cell Killing and Viral Replication

**DOI:** 10.1371/journal.ppat.1003742

**Published:** 2013-11-14

**Authors:** Melissa Z. Mui, Michael Kucharski, Marie-Joëlle Miron, Woosuk Steve Hur, Albert M. Berghuis, Paola Blanchette, Philip E. Branton

**Affiliations:** 1 Department of Biochemistry, McGill University, Montreal, Quebec, Canada; 2 Department of Oncology, McGill University, Montreal, Quebec, Canada; 3 The Rosalind and Morris Goodman Cancer Research Centre, McGill University, Montreal, Quebec, Canada; University of Michigan, United States of America

## Abstract

Adenovirus E4orf4 protein induces the death of human cancer cells and *Saccharomyces cerevisiae*. Binding of E4orf4 to the B/B55/Cdc55 regulatory subunit of protein phosphatase 2A (PP2A) is required, and such binding inhibits PP2A^B55^ activity leading to dose-dependent cell death. We found that E4orf4 binds across the putative substrate binding groove predicted from the crystal structure of B55α such that the substrate p107 can no longer interact with PP2A^B55α^. We propose that E4orf4 inhibits PP2A^B55^ activity by preventing access of substrates and that at high E4orf4 levels this inhibition results in cell death through the failure to dephosphorylate substrates required for cell cycle progression. However, E4orf4 is expressed at much lower and less toxic levels during a normal adenovirus infection. We suggest that in this context E4orf4 largely serves to recruit novel substrates such as ASF/SF2/SRSF1 to PP2A^B55^ to enhance adenovirus replication. Thus E4orf4 toxicity probably represents an artifact of overexpression and does not reflect the evolutionary function of this viral product.

## Author Summary

The adenovirus E4orf4 protein when expressed alone at high levels induces the death of human cancer cells but not normal primary cells. It also is toxic in the yeast *Saccharomyces cerevisiae*, which we have used as a model system in some studies. Toxicity induced by the E4orf4 protein is largely dependent on its ability to associate with the highly conserved B/B55/Cdc55 class of regulatory subunits of protein phosphatase 2A (PP2A), of which the mammalian B55α species is best characterized structurally. We showed previously that binding to B55α appears to inhibit PP2A activity against at least some substrates. In the present study, we mapped the E4orf4 binding site on both yeast Cdc55 and mammalian B55α and propose how such binding may inhibit PP2A activity. The implications of E4orf4 binding on PP2A activity are of significant scientific interest in terms of the process by which PP2A recognizes and dephosphorylates its substrates. We also propose that E4orf4 binding in the context of viral replication serves the quite different function of introducing novel substrates for dephosphorylation by the PP2A holoenzyme.

## Introduction

When expressed alone at high levels E4orf4 protein of human adenovirus induces the selective death of human tumor cells and the yeast *Saccharomyces cerevisiae* [1]–[15]. Killing of cancer cells is p53-independent and resembles apoptosis in some cell lines, but seems to occur by mitotic catastrophe following mitotic arrest in others [2], [4], [6], [10], [12], [13], [16]. Recent observations in a *Drosophila melanogaster* model suggested that E4orf4 induces conflicting signals to apoptotic pathways to influence the type of death response that occurs [17]. Toxicity in yeast is also associated with mitotic arrest [9], [11] and we and others proposed that effects on APC/C lead to abnormal progression through mitosis [9], [14], and in the case of cancer lines accumulation of G1-arrested diploid and tetraploid cells prior to death [13], [18].

Several E4orf4 interacting partners have been identified, including c-Src [6], [16], [17] and the ATP-dependent chromatin-remodeling factor ACF [19], that may contribute to effects induced by E4orf4 expression; however, induction of cell death is highly dependent on interactions with protein phosphatase 2A (PP2A) [5], [7]–[9], [11], [12], [14], [15], [17], [20]–[24]. PP2A is the most abundant Ser/Thr phosphatase, exhibiting extensive pleiotropic activities [25]–[32]. PP2A holoenzymes exist as heterotrimers of a catalytic C subunit, an A subunit scaffold, and a B regulatory subunit that determines intercellular localization and substrate specificity [33]–[36]. About twenty mammalian B subunits exist in three classes designated as B/B55, B′/B56, and B′, as well as B′″ striatin/SG2NA [29], [37]. PP2A of *S. cerevisiae* is highly similar with respect to organization, amino acid sequence, and sensitivity to inhibitors [29]. The catalytic C subunit is encoded by two highly homologous genes, *PPH21* and *PPH22* [38], [39]. *TPD3* encodes the A subunit, which has a structure similar to mammalian A subunits [40]. Only two B-type regulatory subunits exist, encoded by *CDC55* and *RTS1*, which are highly related to the mammalian B/B55 and B′/B56 families, respectively [41], [42].

Products of several small DNA tumor viruses target PP2A either to enhance viral replication or to facilitate cell transformation [43]; however, whereas the interaction of small T antigens of both SV40 and polyoma viruses result in replacement of the B regulatory subunit [44]–[48], the adenovirus E4orf4 protein binds to the complete PP2A holoenzyme. E4orf4 can associate with PP2A holoenzymes through interactions with the B55α regulatory subunit [5], [7]. Although an earlier report suggested some interaction with the B′/B56 class [8], in our studies E4orf4 binding could only be detected with the four members (α, β, γ, and δ) of the B/B55 class [12]. E4orf4 also interacts with Cdc55, the yeast homolog of B55α, but not Rts1 [9], [11], [14], [24]. E4orf4 contains an arginine-rich nuclear and nucleolar targeting sequence [49] that is also critical for binding to B55α [5], [7] and Cdc55 [11]. In yeast, deletion of *CDC55* eliminates much of the E4orf4-induced loss of cell viability [9], [11], [14], [15], [22]. Additionally, in both human tumor cells and yeast, E4orf4 mutants that fail to bind B55α or Cdc55 (termed by our group as class I) are defective in induction of cell death [5], [7], [11], [14].

**Figure 1:**
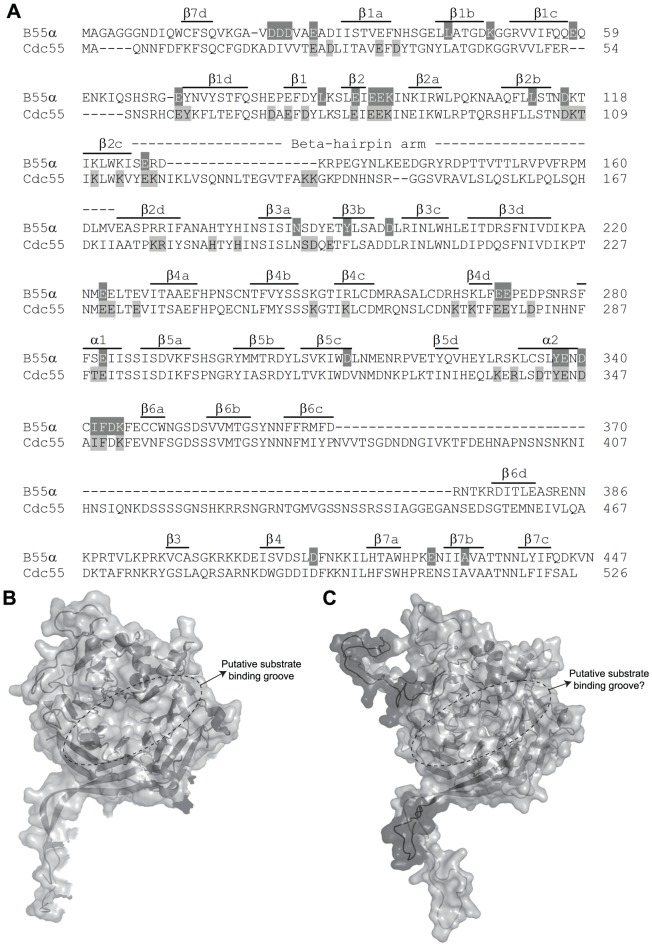
Comparison of B55α and Cdc55. (**A**) Sequence alignment of B55α and Cdc55. Alignment is in relation to the crystal structure of B55α. The positions (or proposed positions for Cdc55) of β-strands within blades 1 to 7 (e.g., β1a, etc.), the β-hairpin loops (b1 to b4), and the β-hairpin arm are indicated above the sequences. Highlighted residues correspond to amino acids subjected to mutational analysis in β55α (top sequence) and Cdc55 (bottom sequence) and are listed in Tables 1 and 2, respectively. (**B**) Known B55α structure shown in ribbon and surface depictions with the putative substrate binding groove of B55α indicated. (**C**) Predicted Cdc55 structure (as generated by ModWeb) shown in ribbon and surface depictions. Highlighted in dark gray are residues that are most divergent from B55α in the primary sequence, as shown in (A).

**Figure 2:**
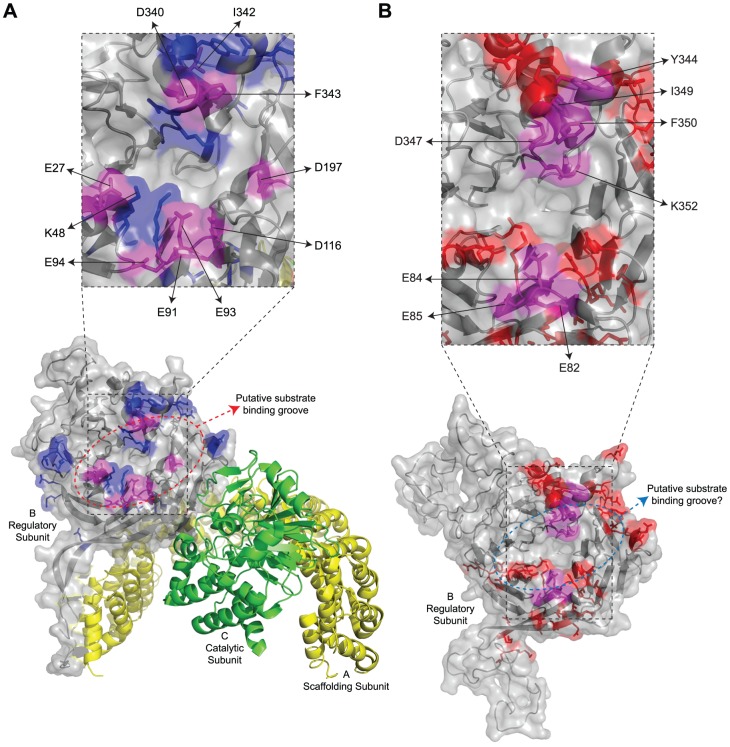
Summary of mutations in mammalian B55α and yeast Cdc55 that affected E4orf4 association. Structure of PP2A^B55α^ and the predicted structure of Cdc55. (**A**) Structure of B55α-containing PP2A holoenzymes (bottom). Highlighted in blue and magenta are all the residues subjected to mutational analyses in this study, where magenta coloured residues indicate the location of mutations where E4orf4 binding was reduced or lost. Residues found to be involved in E4orf4 binding located both *north* and *south* of the putative substrate binding groove are indicated in a zoomed-in panel (top) and listed in Table 4, where mutants are compared to wild type Cdc55. Also indicated are residues E27, K48, and D197 that have been previously shown to be involved in Tau dephosphorylation [50]. PP2A A subunit (yellow), PP2A C subunit (green). (**B**) Predicted structure of Cdc55 (bottom). Highlighted in red and magenta are all the residues subjected to mutational analyses in this study, where magenta coloured residues indicate the location of mutations where E4orf4 binding was reduced or lost. Residues found to be involved in E4orf4 binding located both *north* and *south* of the putative substrate binding groove are indicated in a zoomed-in panel (top) and listed in Table 4.

Figure 1A shows the considerable amino acid similarity in critical parts of Cdc55 and B55α. B55α contains seven WD40 repeats and its resolved crystal structure [50] (Figure 1B) shows that it folds into a seven-bladed β-propeller protein, where each blade is composed of four anti-parallel β-strands (a, b, c and d) (Figure 1A). The crystal structure of B55α-containing PP2A holoenzymes (Figure 2A) revealed that the β-hairpin arm on the bottom face of B55α interacts with the A subunit, and the C subunit binds to the other end through interactions with HEAT repeats 11–15 of the A scaffolding subunit [50], [51]. *In vitro* phosphatase assays using purified PP2A subunits suggested that the top face of B55α possesses a putative acidic substrate binding groove, as mutations affecting residues Glu27, Lys48, and Asp197 decreased phosphatase activity against the substrate Tau [50]. E4orf4 was found to reduce PP2A activity in *in vitro* assays and when expressed at high levels in mammalian cells to induce hyperphosphorylation of certain PP2A substrates [12], [52]. Additionally, low levels of okadaic acid or expression of I_1_^PP2A^, both PP2A inhibitors, actually were found to enhance E4orf4 toxicity [12]. These results suggest that binding of E4orf4 protein inhibits PP2A activity against at least some substrates if sufficiently high levels are expressed and we believe that it is the failure to dephosphorylate substrates necessary for cell cycle progression that induces cell toxicity. The finding that E4orf4 toxicity is tumor cell-specific makes it a potential candidate for development of new cancer therapies [1]–[5], [7], [53]. Thus the establishment of the E4orf4 binding site on B55α/Cdc55 might further our understanding of the mechanism of E4orf4-induced cell death and facilitate development of small molecules that mimic E4orf4 action.

Previous mutational analyses by our group and others to delineate the E4orf4 binding site were initiated before resolution of the B55α crystal structure [21], [22], [24]. Most mutations that affected E4orf4 binding were located within the β-sheets of the propeller structure and thus likely to affect the intricate spacing of the β-propeller structure of B/B55 subunits [24]. With the present knowledge of the detailed structure of B55α [50] we revisited the possibility of identifying the E4orf4 binding site on both Cdc55 and B55α by introducing more meaningful mutations located on exposed surfaces. Using this approach we delineated regions of both Cdc55 and B55α involved in E4orf4 binding. In both cases E4orf4 binding occurs across the putative substrate binding groove, and with B55α, E4orf4 was shown to prevent binding and dephosphorylation of the substrate p107, suggesting that inhibition of PP2A activity may result from interference with the access of substrates to the holoenzyme. Nonetheless, we believe that the major function of E4orf4 during viral replication, wherein E4orf4 levels are considerably lower, is to target substrates such as ASF/SF2/SRSF1 to PP2A^B55^ to enhance their dephosphorylation.

## Results

### Generation of Cdc55 and B55α mutants

As previous mutational analyses of both yeast Cdc55 and mammalian B55α to delineate the E4orf4 binding site were initiated prior to elucidation of the B55α crystal structure [21], [22], [24], we embarked on a new study using a more meaningful array of mutants. Although the crystal structure for Cdc55 has not yet been determined, Figure 1A shows that approximately 56% identity and a high degree of similarity exists between the orthologs, except for two regions unique to Cdc55, located between β2c and β2d, and β6c and β6d. Figures 1B and 1C illustrate the structure of B55α and predicted structure of Cdc55, respectively, where dark gray areas on Cdc55 indicate two regions absent in B55α but distant from the predicted substrate binding pocket. Differences entail an extended loop in the region where the β-hairpin arm should be located, as well as an additional loop not present in B55α that protrudes from the bottom face of Cdc55 (Figures 1B and 1C). The predicted Cdc55 structure also exhibits a putative substrate binding groove similar to that of B55α. We used site-directed mutagenesis to produce a series of mutants to map the E4orf4 binding site on both Cdc55 and B55α, targeting residues that were conserved between B55α and Cdc55 and present in exposed surfaces. All residues targeted in this study are shown in Figure 1A, and all mutations listed in Tables 1 (B55α) and 2 (Cdc55).

**Table 1:** B55α residues subjected to point mutations.

B55α Point Mutations:	
D22K	E223K
D23K	E270K
D24K	E271K
E27K	E283K
D22K/D23K/D24K/E27K	Y337A
E27R	Y337F
K48R	E338K
E27K/K48R	D340A
E58K	D340K
E70K	I342A
L87A	E91A/E93A/E94A/I342A
E91K	F343A
E93K	E27R/F343A
E27K/E93K	K48E/F343A
E94K	E91A/E93A/E94A/F343A
K95D	D116K/F343A
E91A/E93A/E94A	D197K/F343A
E91K/E93K/E94K	D340A/F343A
E91A/E93A/E94A/K95A	I342A/F343A
D116K	D344K
E126K	K345A
R127E	K345D
Y192A	D340A/K345A
D197K	I342A/F343A/K345A
E27K/D197K	D414K
E27K/K48R/D197K	A432L
F343A/D197K	Y192A/A432L

**Table 2:** Cdc55 residues subjected to point mutations.

Cdc55 Point Mutations:
E24A/D26A
E32A/D34A
E61A/Y62A
D72A/E74A/D76A
E82A
E84A
E85A
K86A
E82A/E84A/E85A/K86A
E82K
E84K
E85K
K86E
E82K/E84K/E85K/K86E
D107A/K108A/T109A
K111A/K114A
E117A/K118A
K136A/K137A
K176A/R177A
H183A/H186A
S194A/D195A/E197A
E230A/E231A/E234A
K255A/K259A
K272A/K274A
E277A/E278A/D281A
T289A/E290A
K337A/R339A
D342A
E345A
D347A
D342A/E345A/D347A
D342K
E345K
D347K
D342K/E345K/D347K
Y344A
Y344F
I349A
F350A
K352A

### Residues in both B55α and Cdc55 required for E4orf4 binding are located *north* of the putative substrate binding groove

**Figure 3:**
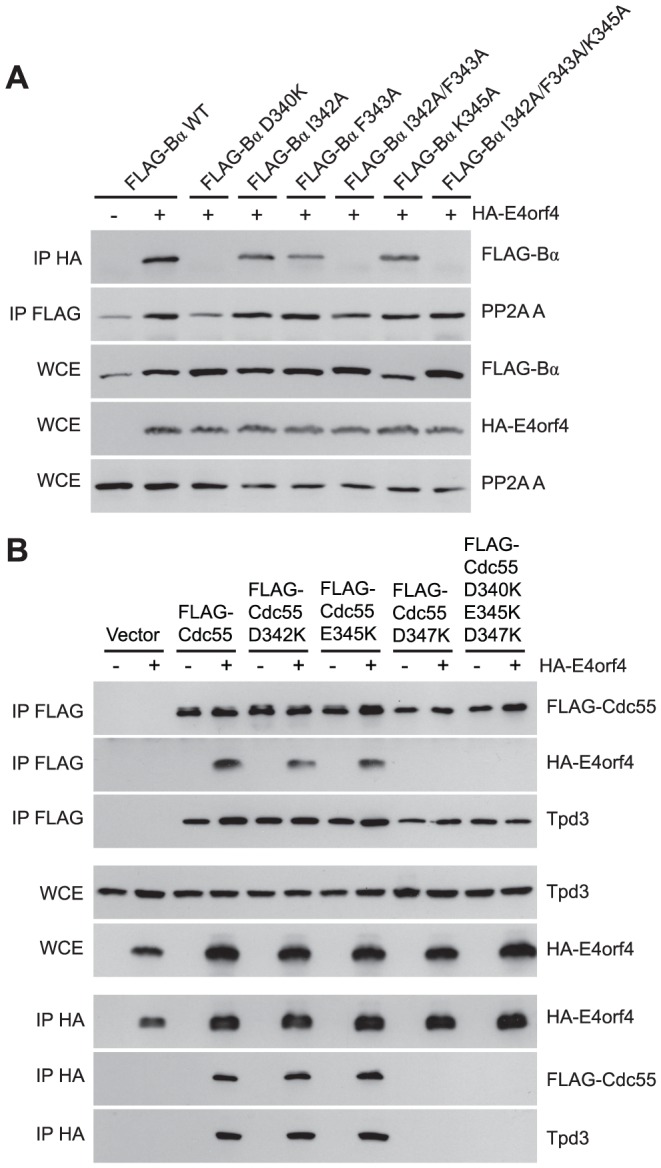
Mutations *north* of the substrate binding groove affect E4orf4 association with B55α and Cdc55. E4orf4 association with mammalian B55α and yeast Cdc55 mutants affecting regions *north* of the putative substrate binding groove. (**A**) Co-immunoprecipitation experiments where HA-E4orf4 was immunoprecipitated using anti-HA antibodies and western blots performed using anti-FLAG antibodies to detect binding of wild type FLAG-B55α (B55α) to mutant species (D340K, I342A, F343A, I342A/F343A, K345A, and I342A/F343A/K352A). All mutant B55α species were immunoprecipitated with anti-FLAG antibodies and western blots performed using anti-PP2A A antibodies to detect the PP2A holoenzyme-forming abilities of B55α mutants shown. Whole cell extracts (WCE) shown for FLAG-B55α species, HA-E4orf4, and PP2A A scaffolding subunit. (**B**) Co-immunoprecipitation experiments where wild type FLAG-Cdc55 or mutant species (D342K, E345K, D347K, and D342K/E345K/D347K) were immunoprecipitated using anti-FLAG antibodies and western blots performed using anti-HA antibodies to detect HA-E4orf4 binding (top). Reciprocal co-immunoprecipitation experiments where HA-E4orf4 was immunoprecipitated using anti-HA antibodies and western blots performed using anti-FLAG antibodies to detect binding of wild type or mutant FLAG-Cdc55 to HA-E4orf4 (bottom). Co-immunoprecipitation between wild type or mutant Cdc55 species and the scaffolding A subunit Tpd3 was assessed to determine whether the mutation altered the global protein structure. Whole cell extracts (WCE) shown for HA-E4orf4 and Tpd3 (middle).

Although many B55α mutants were generated and all rigorously tested, only data pertaining to mutations affecting E4orf4 binding will be discussed in detail. Mutations specified in Figure 1A and Table 1 were made in FLAG-B55α-expressing plasmid DNAs that were then transfected into H1299 cells along with HA-E4orf4-expressing plasmid DNAs to test E4orf4 binding. Results from co-immunoprecipitation experiments with protein extracts from these cells are shown in Figure 3A, where HA-E4orf4 was immunoprecipitated using anti-HA antibodies and western blotting performed using anti-FLAG antibodies to detect binding to FLAG-B55α species. Unlike the binding of wild type B55α to E4orf4, a charge reversal of Asp340 to Lys completely eliminated E4orf4 binding. Mutation of nearby Ile342 and Phe343 to Ala residues individually had either no effect or exhibited reduced binding, respectively; however, in combination the I342A/F343A mutant was totally defective in E4orf4 binding. Conversion of nearby Lys345 to Ala did not affect binding greatly; however, in combination the I342A/F343A/K345A mutant was totally defective. In studies such as these it is never certain that alteration of even a single residue might induce a significant change in overall protein conformation; however, results from co-immunoprecipitation experiments showed that all mutants were still capable of associating with the PP2A A subunit, suggesting that little overall change in structure occurred. Thus Asp340 and Phe343 appear to be important for E4orf4 binding. These residues mapped to a helical region that protrudes from the top face of the main β-propeller structure (Figure 2A). Interestingly, this region is located just “north” of the putative substrate binding groove, where *north* is defined relative to the orientation of B55α shown in Figure 2A, and where the substrate binding groove runs “east-west” on the top face of B55α/Cdc55, with *east* located near the catalytic subunit.

Mutants were also generated in Cdc55, and again, only data pertaining to mutations that had significant effects on E4orf4 binding will be discussed. Mutations, as specified in Figure 1A and Table 2, were made and plasmid DNAs expressing altered Cdc55 proteins were transformed into yeast cells. A strain lacking *CDC55* was used, to yield yeast cells exogenously expressing FLAG-tagged versions of wild type or mutant Cdc55, in the presence or absence of E4orf4 expressed from a second plasmid DNA. Figure 3B shows results from an experiment in which Cdc55 mutants were assessed for E4orf4 and A subunit binding in reciprocal co-immunoprecipitation studies. Cdc55 mutants involving reversal of negative charge on Asp342, Glu345, and Asp347 individually, or in combination, were tested. Of the three, D347K had a pronounced effect relative to wild type Cdc55, and the D342K/E345K/D347K triple mutant was also incapable of binding E4orf4, even though all were capable of interacting with Tpd3. Alteration of these residues to Ala yielded similar results (data not shown). It should be noted that D347K is the yeast equivalent to D340K in B55α shown in Figure 3A that failed to bind E4orf4. Placement of Asp347 on the predicted structure of Cdc55 is shown in Figure 2B.

To correlate E4orf4 binding with toxicity, cell growth assays were conducted in the presence of either empty or E4orf4-expressing plasmid DNAs using *cdc55Δ* background yeast strains exogenously expressing Cdc55 *north* mutants under the normal *CDC55* promoter. Table 3 summarizes cell growth assays as well as PP2A holoenzyme-forming capabilities of the Cdc55 mutants, where all mutants are compared to wild type Cdc55 and representative data are shown in Figure S1. All mutants were capable of reconstituting rapamycin sensitivity like wild type Cdc55, supporting the observation that all were capable of interacting with Tpd3 in Figure 3B. The ability of Cdc55 *north* mutants to bind E4orf4 closely correlated with E4orf4-induced cell death. E4orf4 was toxic or partially toxic in cells expressing the D342K and E345K mutants, respectively, both of which were able to associate with E4orf4; however, in cells expressing D347K and D342K/E345K/D347K that were unable to bind E4orf4, little toxicity was observed. Taken together, these results suggested that E4orf4 binds to comparable helical regions on both Cdc55 and B55α that protrude from the top face of the main β-propeller structure located just *north* of the putative substrate binding groove, and that this binding correlates with the ability of E4orf4 to induce cell death.

**Table 3:** Summary of cell growth assays with Cdc55 mutants.

Cdc55 Point Mutations	Ability to Bind E4orf4	Rescue From Toxicity	Rapamycin Sensitivity	Location of Mutation
WT	++++	−	++++	n/a
D342K	++++	−	++++	north
E345K	++++	+	++++	north
D347K	−	++	++++	north
D342K/E345K/D347K	−	++	++++	north
E82K	++	++	++++	south
E84K	+	++	++++	south
E85K	++	+++	++++	south
K86E	++++	++++	++++	south
E82K/E84K/E85K/K86E	−	++	++++	south

### Residues in both B55α and Cdc55 *south* of the substrate binding groove are also involved in E4orf4 binding

**Figure 4:**
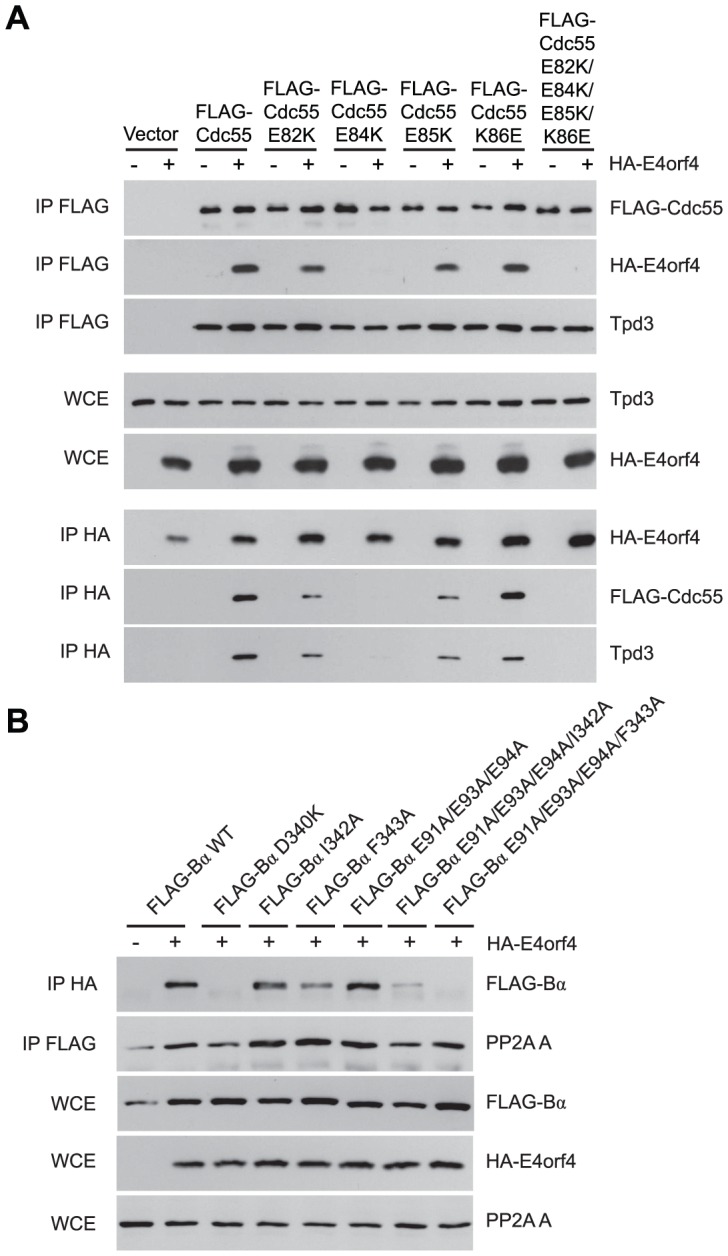
Mutations *south* of the substrate binding groove also affect E4orf4 association with B55α and Cdc55. E4orf4 association with mammalian B55α and yeast Cdc55 mutants affecting regions *south* of the putative substrate binding groove. (**A**) Co-immunoprecipitation experiments similar to those shown in Figure 3B where wild type FLAG-Cdc55 or mutant species (E82K, E84K, E85K, K86E and E82K/E84K/E85K/K86E) were immunoprecipitated using anti-FLAG antibodies and western blots performed using anti-HA antibodies to detect binding of HA-E4orf4 (top). Reciprocal co-immunoprecipitation experiments where HA-E4orf4 was immunoprecipitated using anti-HA antibodies and western blots performed using anti-FLAG antibodies to detect binding of wild type or mutant FLAG-Cdc55 to HA-E4orf4 (bottom). Co-immunoprecipitation between wild type and mutant Cdc55 species and the A subunit Tpd3 was assessed to determine whether the mutation altered the global protein structure. Whole cell extracts (WCE) shown for HA-E4orf4 and Tpd3 (middle). (**B**) Co-immunoprecipitation experiments similar to those shown in Figure 3A where HA-E4orf4 was immunoprecipitated using anti-HA antibodies and western blots performed using anti-FLAG antibodies to detect binding of wild type FLAG-B55α or mutant species (D340K, I342A, F343A, E91A/E93A/E94A, E91A/E93A/E94A/I342A, and E91A/E93A/E94A/F343A). All mutant B55α species were immunoprecipitated with anti-FLAG antibodies and western blots performed using anti-PP2A A antibodies to detect the PP2A holoenzyme-forming abilities of B55α mutants. Whole cell extracts (WCE) shown for FLAG-B55α species, HA-E4orf4, and PP2A A scaffolding subunit.

Additional residues involved in E4orf4 binding located *south* of the putative Cdc55 substrate binding groove were identified using further Cdc55 mutants involving charge reversals of Glu82, Glu84, Glu85 and Lys86, either individually or in combination. Figure 4A shows that although all mutants were still able to bind Tpd3, E82K and E85K bound E4orf4 at slightly reduced levels and K86E was similar to wild type Cdc55; however, E84K and the combined mutant were completely deficient. Similar results were obtained with Ala substitutions (data not shown).

Cell growth assays were performed with these mutants, with results again summarized in Table 3 and representative data shown in Figure S2. All mutants were capable of reconstituting rapamycin sensitivity. E4orf4 was partially toxic in cells expressing E82K that was partially defective in E4orf4 binding. E4orf4 exhibited reduced toxicity with E84K and E82K/E84K/E85K/K86E that failed to interact stably with E4orf4. E4orf4-induced toxicity was low with mutants E85K and K86E, despite the fact that they were partially defective or apparently normal for E4orf4 binding, respectively. Thus unlike the Cdc55 *north* mutants, the ability of *south* mutants to bind E4orf4 poorly correlated with E4orf4 toxicity.

Taken together, these results suggested that this region of Cdc55 is also implicated in E4orf4 binding. Placement of Glu82, Glu84, and Glu85 on the predicted Cdc55 structure is shown in Figure 2B, indicating a region *south* of the putative substrate binding groove directly across from the helical region previously found to be involved in E4orf4 binding.

Because a region *south* of the Cdc55 putative substrate binding groove was implicated in E4orf4 binding, studies were conducted to determine if this region also plays a role with mammalian B55α. Corresponding mutations were generated and introduced into B55α-expressing plasmid DNAs and tested for E4orf4 and PP2A A binding as in Figure 3A. Figure 4B shows that unlike the situation in yeast, B55α mutant E91A/E93A/E94A appeared normal for E4orf4 binding; however, all or almost all binding was lost when these amino acid alterations were combined with *north* mutations, namely I342A and F342A that alone exhibited wild type or only partially reduced binding, respectively. All mutants formed PP2A holoenzymes as all were capable of co-immunoprecipitating with the A subunit. These results suggested that this region of B55α *south* of the putative substrate binding groove is also implicated in E4orf4 binding (Figure 2A).

Taken together, these results suggested that the interaction of E4orf4 with the *north* region of B55α is much stronger than with the region to the *south*. They also suggested that with Cdc55 a significant interaction with E4orf4 takes place on both sides of the putative substrate binding groove. These differences in binding may not be surprising and the fact that E4orf4 binds both efficiently and functionally to Cdc55 reflects a remarkable conservation of structure from yeast to man. And importantly, the mutational analyses in yeast were invaluable, as it may not otherwise have been possible to implicate the *south* region of B55α in binding.

### The E4orf4 and Tau substrate binding sites partially overlap

**Figure 5:**
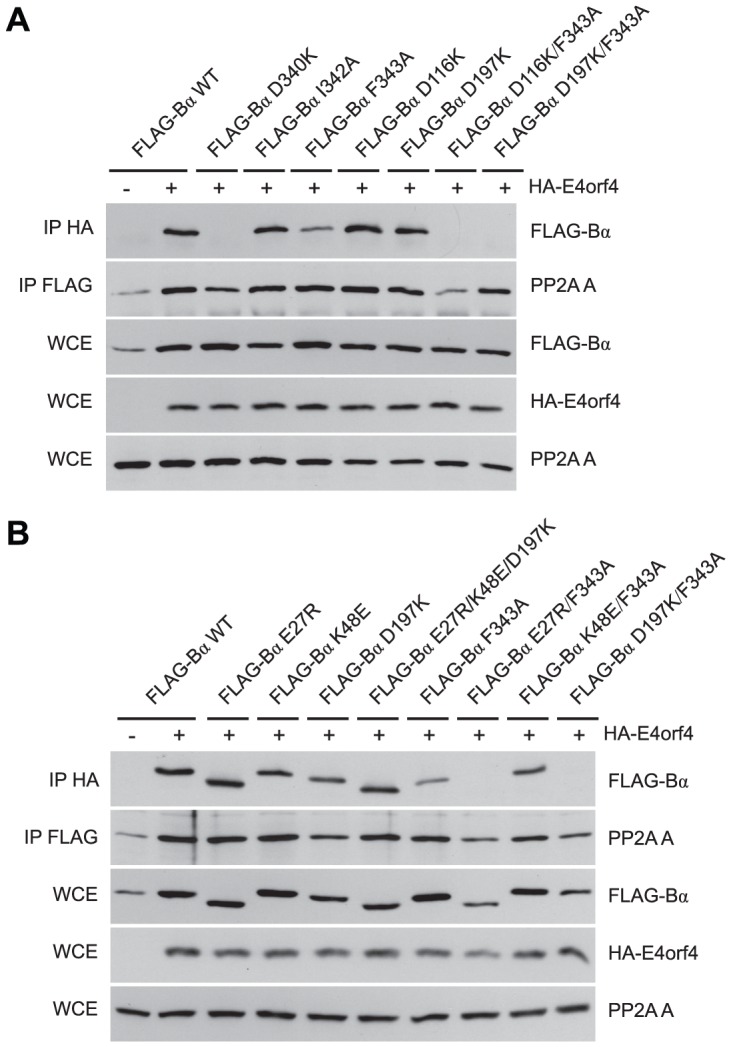
B55α residues involved in Tau dephosphorylation and E4orf4 binding. Studies to relate E4orf4 binding to that of the Tau substrate. (**A**) Co-immunoprecipitation experiments similar to those in Figures 3 and 4 where HA-E4orf4 was immunoprecipitated using anti-HA antibodies and western blots performed using anti-FLAG antibodies to detect binding of wild type FLAG-B55α to mutant species (D340K, I342A, F343A, D116K, D197K, D116K/F343A, and D197K/F343A). All mutant B55α species were immunoprecipitated with anti-FLAG antibodies and western blots performed using anti-PP2A A antibodies to detect the PP2A holoenzyme-forming abilities. Whole cell extracts (WCE) shown for FLAG-B55α species, HA-E4orf4, and PP2A A scaffolding subunit. (**B**) Co-immunoprecipitations were performed as in (A) with wild type FLAG-B55α or mutant species (E27R, K48E, D197K, E27R/K48E/D197K, F343A, E27R/F343A, K48E/F343A, and D197K/F343A). Residues E27, K48, and D197 have been previously shown to be involved in the dephosphorylation of Tau [50].

To characterize E4orf4 binding further, additional B55α mutants were tested as before. D116K and D197K, the latter previously proposed to be implicated in binding of the Tau substrate [50], alone were capable of associating normally with E4orf4 (Figure 5A); however, binding was lost when these alterations were combined with the F343A *north* mutation, which alone exhibited only slightly reduced binding. Again, co-immunoprecipitation experiments involving the PP2A A subunit showed that the overall structure of the B55α mutants was mostly intact, as all mutants were capable of associating with the PP2A A subunit. These results suggested that both Asp116 and Asp197 of B55α are also implicated in interactions with E4orf4. The remaining residues proposed to be involved in Tau binding [50] were also modified and mutants assessed for E4orf4 binding. Figure 5B shows that as with D197K, E27R and K48E alone were able to bind E4orf4 normally, as was the case with a mutant combining alterations in all three residues involved in Tau dephosphorylation (E27R/K48E/D197K); however, as previously seen when the D197K mutation was combined with the F343A *north* mutation, the combined mutant E27R/F343A also lost the ability to bind E4orf4. Interestingly, the combined mutant K48E/F343A exhibited no additional loss of E4orf4 binding when compared to F343A alone. These results suggested that there is considerable but not complete overlap in the binding sites of E4orf4 and the PP2A substrate Tau (see Figure 2A).

### Binding of class II E4orf4 mutants differs from wild type

**Figure 6:**
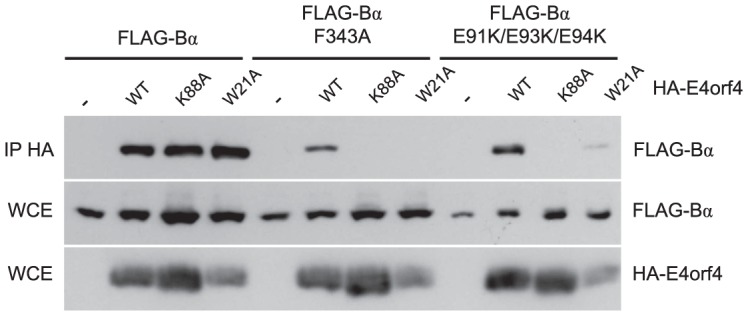
Binding of class II E4orf4 mutants to B55α mutants. Co-immunoprecipitation experiments where HA-E4orf4 (WT) or class II mutants K88A and W21A were immunoprecipitated using anti-HA antibodies and western blots performed using anti-FLAG antibodies to detect binding of wild type FLAG-B55α or mutant species (F343A or E91K/E93K/E94K). Whole cell extracts (WCE) shown for HA-E4orf4 and FLAG-B55α species.

We previously described two classes of E4orf4 mutants. Class I are unable to bind to B55α/Cdc55 and induce significantly reduced toxicity. Class II bind B55α/Cdc55, but nonetheless have reduced toxicity, suggesting that binding is not sufficient to exert a biological effect [7], [11]. To determine if differences in binding to B55α could explain the class II phenotype we examined binding of representative class II E4orf4 mutants K88A and W21A [7] to B55α species bearing both *north* (F343A) and *south* (E91K/E93K/E94K) mutations. F343A was chosen as it was partially defective in wild type E4orf4 binding (Figure 3A) whereas the E91/E93/E94 *south* B55α mutant exhibited normal levels of binding of wild type E4orf4 when the residues were converted to alanines (Figure 4B). HA-E4orf4 proteins (wt, K88A and W21A) were co-expressed with FLAG-B55α (wild type, F343A, and E91K/E93K/E94K) in H1299 cells and binding assays were conducted. Figure 6 shows that whereas both class II mutants interacted well with wild type B55α, both exhibited greatly reduced binding to *north* (F343A) and *south* (E91K/E93K/E94K) mutant B55α species. These results demonstrated a clear difference in the interaction of wild type and class II E4orf4 proteins with B55α.

### E4orf4 prevents binding of the PP2A^B55α^ substrate p107

**Figure 7:**
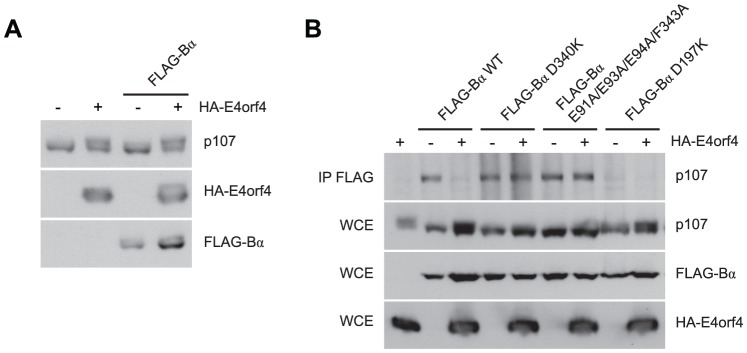
E4orf4 causes p107 hyperphosphorylation and prevents binding of p107 to PP2A^B55α^. Effects of E4orf4 on PP2A^B55α^ substrate p107. (**A**) Whole cell extracts (WCE) prepared from cells transfected with HA-E4orf4 and FLAG-B55α and western blots performed using anti-p107, anti-FLAG, and anti-HA antibodies. (**B**) Co-immunoprecipitation experiments where wild type FLAG-B55α or mutant species (D340K, E91A/E93A/E94A/F343A, and D197K) were immunoprecipitated using anti-FLAG antibodies and western blots performed using anti-p107 antibodies. WCE shown for HA-E4orf4, FLAG-B55α species, and p107.

Our results suggested that E4orf4 binds across the putative B55α substrate binding groove and thus access of substrates may be obstructed (Figure 2A). One known PP2A^B55α^ substrate is p107, a member of the Rb pocket protein family [54]–[56]. Figure 7A shows that expression of HA-E4orf4 in the absence and presence of exogenous FLAG-B55α results in hyperphosphorylation of p107, as indicated by reduced p107 mobility on SDS-PAGE. To test the effect of E4orf4 on p107 binding to B55α, co-immunoprecipitation experiments were conducted using wild type B55α, two B55α mutants that no longer bind to E4orf4 (D340K and E91A/E93A/E94A/F343A), as well as another B55α mutant previously shown to no longer bind to p107 (D197K) [54]. These B55α species were expressed in H1299 cells in the absence and presence of E4orf4. Figure 7B shows that wild type B55α interacted with p107; however, in the presence of E4orf4, this interaction was greatly reduced. With B55α mutants D340K and E91A/E93A/E94A/F343A, p107 binding occurred at high levels regardless of the presence or absence of E4orf4. The B55α D197K mutant failed to bind p107, and this effect did not change in the presence of E4orf4. Although simple binding assays such as these might not reflect activity against p107, these results do strongly support our hypothesis that, at least in the case of some substrates, E4orf4 inhibits PP2A activity by preventing access to the holoenzyme.

### Role of E4orf4 in viral infection: E4orf4 targets ASF/SF2/SRSF1 to PP2A^B55α^

**Figure 8:**
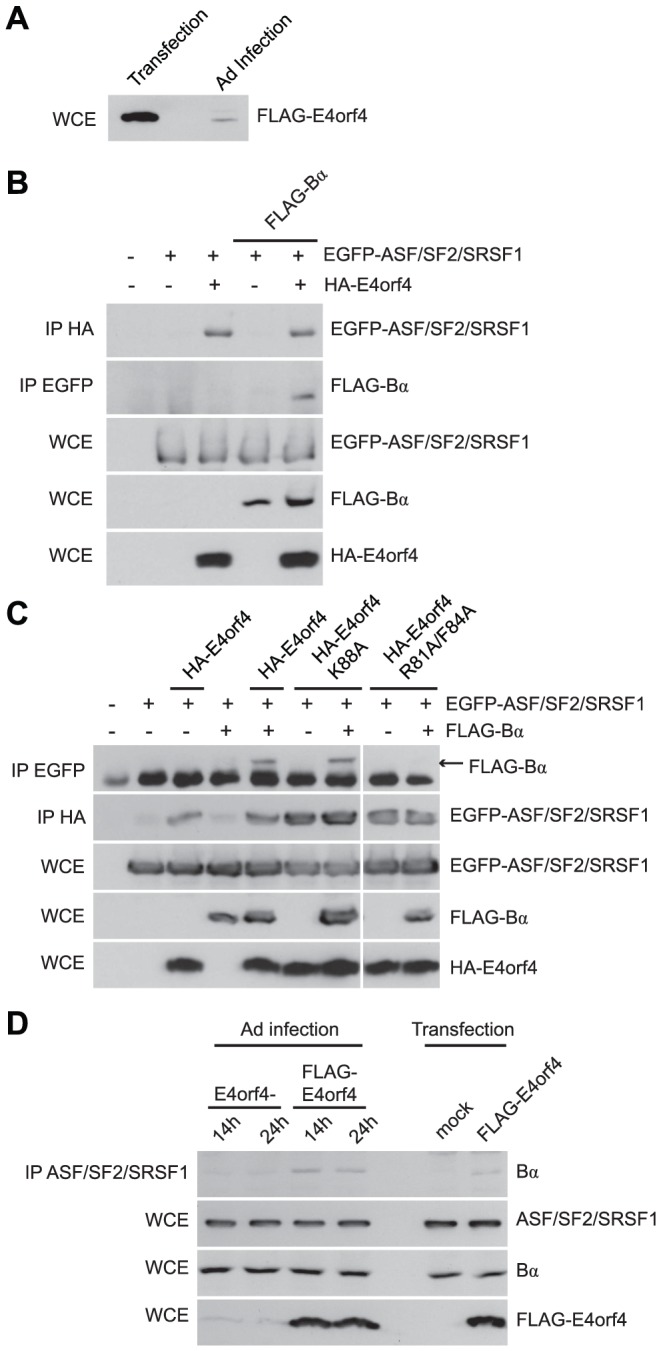
E4orf4 targets ASF/SF2/SRSF1 to PP2A^B55α^. Role of E4orf4 in adenovirus infection. (**A**) SDS-PAGE analysis comparing the expression levels of FLAG-E4orf4 by plasmid DNA transfection (5 µg of protein extract loaded) and adenovirus infection at a multiplicity of infection (MOI) of 5 with Ad5 FLAG-E4orf4 virus of H1299 cells (100 µg of protein extract loaded). (**B**) Co-immunoprecipitation experiments where HA-E4orf4 was immunoprecipitated using anti-HA antibodies and western blots performed using anti-EGFP antibodies to detect interactions between HA-E4orf4 and EGFP-ASF/SF2/SRSF1. Additionally, EGFP-ASF/SF2/SRSF1 was immunoprecipitated using anti-EGFP antibodies and western blots performed using anti-FLAG antibodies to detect interactions between EGFP-ASF/SF2/SRSF1 and FLAG-B55α. Whole cell extracts (WCE) shown for EGFP-ASF/SF2/SRSF1, FLAG-B55α, and HA-E4orf4. (**C**) Similar co-immunoprecipitation experiments were performed as in (B) except with the addition of class II (HA-E4orf4 K88A) and class I (HA-E4orf4 R81A/F84A) E4orf4 mutants. (**D**) Co-immunoprecipitation experiments where ASF/SF2/SRSF1 was immunoprecipitated using anti-ASF antibodies and western blots performed using anti-B55α antibodies to detect interactions between ASF/SF2/SRSF1 and B55α during the course of infection (MOI = 20) with virus lacking E4orf4 (E4orf4-) and wild type Ad5 FLAG-E4orf4 (14 h and 24 h), as well as FLAG-E4orf4 transfection. All loadings for WCE were equal except for FLAG-E4orf4 where 100 µg of protein extract was loaded in samples involving infection and 25 µg for those of transfection.

We have been aware for some time that induction of cell toxicity may not represent the true role of E4orf4 during adenovirus infection. E4orf4 killing is dose-dependent [7], [12] and Figure 8A shows by western blotting against FLAG that the level of E4orf4 expression during a typical infection with wild type Ad5 expressing FLAG-E4orf4 [57] is more than an order of magnitude lower than that required for efficient cell death in routine killing assays where E4orf4 is expressed via plasmid DNA or viral vector. Thus other E4orf4 functions may be of much more importance to the virus. E4orf4 is known to facilitate the switch in viral mRNA expression from early to late genes [58] and was found to interact with the SR proteins ASF/SF2/SRSF1 and SRp30c. Furthermore, E4orf4 point mutants that no longer associate with either PP2A or ASF/SF2/SRSF1 were found incapable of relieving the repressive effect of SR proteins on splicing of late adenoviral mRNA. These results suggested that the dephosphorylation of ASF/SF2/SRSF1 by PP2A alters splice site selection to allow late viral protein production [59]. Thus the actual role of E4orf4 in the infectious cycle may be to deliver critical substrates to B55-containing PP2A holoenzymes, including ASF/SF2/SRSF1, as it has been found to be an E4orf4 interacting partner (Teodoro, J.G., unpublished results; [59]).

Thus we performed co-immunoprecipitation experiments using EGFP-ASF/SF2/SRSF1, HA-E4orf4, and FLAG-B55α. Figure 8B shows that an interaction between E4orf4 and ASF/SF2/SRSF1 was detected, as found previously [59]; however, we also observed that an interaction between B55α and ASF/SF2/SRSF1 occurred only in the presence of E4orf4, suggesting that E4orf4 targets ASF/SF2/SRSF1 to B55α-containing PP2A holoenzymes. Figure 8C shows that whereas B55α associated with ASF/SF2/SRSF1 in the presence of wild type E4orf4 or the class II E4orf4 mutant K88A, no such association was seen with the class I mutant R81A/F84A. These results were in agreement with a previous report suggesting that an active PP2A complex is required for E4orf4-induced SR protein dephosphorylation and late viral mRNA splicing [59]. Additionally, we performed co-immunoprecipitation experiments to determine interactions between endogenous ASF/SF2/SRSF1 and B55α during adenovirus infection. Figure 8D shows that B55α was only capable of interacting with ASF/SF2/SRSF1 in the presence of FLAG-E4orf4. Moreover, during the course of a 24 h infection with wild type Ad5 expressing FLAG-E4orf4, the interaction between ASF/SF2/SRSF1 and B55α was enhanced compared to a similar infection involving adenovirus lacking E4orf4. These results suggest that during adenovirus infection, ASF/SF2/SRSF1 is targeted to the B55α form of PP2A in an E4orf4-dependent manner.

## Discussion

Our previous studies suggested that E4orf4 binding to the regulatory subunit B55α inhibits PP2A activity against at least some substrates [12]. To determine the molecular basis for this inhibition we used a genetic approach to elucidate E4orf4 binding sites. Figures 2A and 2B summarize all mutations made in both B55α and Cdc55, respectively. The magenta highlighted residues on the B55α structure (listed in Table 4 with their E4orf4 binding abilities relative to wild type B55α) in the PP2A^B55α^ holoenzyme (Figure 2A) indicate the location of mutations that reduced or eliminated E4orf4 binding. Similarly, indicated on the predicted Cdc55 structure (Figure 2B) are magenta highlighted residues (listed in Table 4 with their E4orf4 binding abilities relative to wild type Cdc55), which represent sites on Cdc55 to which E4orf4 binds. Our current results suggest that E4orf4 binds to a distinct helical region of both B55α and Cdc55 located on the *north* side of the putative substrate binding groove (Figure 2). This region of B55α was also identified in a recent study published while the present report was being prepared [23]. The Phe343 residue identified by Horowitz *et al.* as being involved in E4orf4 binding was the only residue in common with our current study. Although this group identified Tyr337 as being involved in E4orf4 binding, in our hands this same residue converted to either Ala or Phe showed no loss of E4orf4 binding (Table 1); however, both studies pointed to the helical region *north* of the putative substrate binding groove as a region of interaction for E4orf4. Horowitz *et al.* also implicated an adjacent helix region containing Phe280. We did not find this particular helix to be involved in E4orf4 binding as alteration of Glu283 had no effect. Nevertheless we did not systematically examine this helix and thus the E4orf4 *north* binding site on B55α may be broader than that proposed in our study. But importantly, in addition to this *north* region identified by both groups, we found that a region in both B55α and Cdc55 directly across and just *south* of the putative substrate binding groove is also implicated in E4orf4 binding (Figure 2). Amino acid alterations in this region had a greater effect with Cdc55 than with B55α as mutations in this area of Cdc55 alone were sufficient to eliminate interactions with E4orf4 (Figure 4A) whereas combinatorial mutations were necessary with B55α (Figure 4B). These findings were not surprising as there are minor differences between the B55α structure and the predicted Cdc55 structure in this region (Figure 1 and Figure 2) and clearly E4orf4 has evolved to associate with mammalian B55α not Cdc55. Nevertheless, binding of E4orf4 to corresponding regions of both Cdc55 and B55α appears to exert similar downstream biological effects in yeast and mammalian cells in terms of toxicity and cell cycle perturbations [9], [11]–[14].

**Table 4:** Summary of B55α and Cdc55 mutants that affected E4orf4 binding.

Protein	Point Mutations	Ability to Bind E4orf4
B55α	WT	++++
	D340K	−
	E91A/E93A/E94A/I342A	++
	F343A	++
	E91A/E93A/E94A/F343A	−
	E27R/F343A	−
	D116K/F343A	+
	D197K/F343A	−
	D340A/F343A	−
	I342A/F343A	−
	I342A/F343A/K345A	−
Cdc55	WT	++++
	E82A	+++
	E84A	++
	E85A	+++
	E82A/E84A/E85A/K86A	−
	E82K	++
	E84K	+
	E85K	++
	E82K/E84K/E85K/K86E	−
	D347A	−
	D342A/E345A/D347A	−
	D347K	−
	D342K/E345K/D347K	−
	Y344A	++
	Y344F	++
	I349A	−
	F350A	−
	K352A	++

Correlation of E4orf4 binding with toxicity in yeast (Table 3, and Figures S1 and S2) indicated that although a high degree of association was found between the E4orf4 binding and toxicity with Cdc55 *north* mutants, this correlation was less perfect with some of the Cdc55 *south* mutants. Perhaps such minor discrepancies, particularly with the K86E Cdc55 mutant, are comparable to class II E4orf4 mutants, which, despite being able to associate with B55α, are defective in cell killing [7]. As shown in Figure 6, class II E4orf4 mutants bind to B55α in a very unstable manner as may be the case for E4orf4 binding to the K86E Cdc55 mutant. The observation that some Cdc55 mutants that co-immunoprecipitated less well with E4orf4 were still partially able to transduce E4orf4 toxicity resembles results obtained by Horowitz *et al.* with B55α in very different analyses in which, using nuclear condensation and fragmentation as a measure of cell killing, they demonstrated only a small statistical reduction in toxicity even with *north* B55α mutants defective in E4orf4 binding [23].

Our data strongly suggest that E4orf4 binding to B/B55 subunits traverses the putative substrate binding groove and subsequently may interfere with interactions of some or all substrates (Figure 2). The distance between the regions *north* and *south* of the putative substrate binding groove of B55α is between 15 to 25 Å, a distance that could potentially accommodate a single E4orf4 protein molecule. Although the structure of E4orf4 has not been determined, it is predicted to be composed of three helices [23] that could potentially bind to Cdc55 or B55α across the substrate binding groove. Our group and others have found that E4orf4 expression correlates with reduced PP2A activity against at least some potential PP2A^B55α^ substrates such as p70^S6K^ and 4E-BP1 [12], [52], and we hypothesized that E4orf4 induces cell death by blocking the ability of PP2A^B55^ to dephosphorylate key substrates. We now have strong evidence in support of this hypothesis as we demonstrated that E4orf4 inhibits the binding of substrate p107 to PP2A^B55α^ (Figure 7). These results also validated the functional role of the substrate binding groove proposed for this region in a previous analysis of B55α [50]. Two of three residues shown previously to be implicated in Tau dephosphorylation, Glu27 and Asp197, appear to be involved in binding E4orf4, although the third, Lys48, was not (Figure 5B), thus suggesting a partial overlap. Interestingly, Asp197 of B55α was previously found to be involved in p107 binding [54], an observation confirmed in our studies (Figure 7B). Taken together, these findings suggest that there may be an important overlap for interactions between B55α and its substrates and with E4orf4. We have attempted to conduct similar binding studies with Tau and certain other putative PP2A^B55α^ substrates; however, we were not able to find conditions to detect stable interactions. It is possible that p107 may be retained more readily than most substrates because it is multiply phosphorylated. The fact that the E4orf4 binding site on B55α does not completely conform to the putative substrate binding site allows the possibility that binding of some substrates to PP2A^B55α^ holoenzymes could still occur in the presence of E4orf4. Nevertheless E4orf4 could serve as a unique tool in identifying at least some as yet unidentified PP2A^B55^ substrates.

Several scenarios by which E4orf4 could modify PP2A activity are possible. In addition to inhibiting PP2A activity, E4orf4 could target substrates to the PP2A holoenzyme, or E4orf4 binding could even modify the substrate binding groove such that it could accommodate new substrates; however, we believe that these functions are probably not crucial for the selective killing of human tumor cells. Inhibition of PP2A activity actually enhances toxicity in mammalian tumor cells [12], suggesting that PP2A phosphatase activity per se is not important for toxicity. Our model is that when overexpressed, E4orf4 titrates out the pool of functional PP2A^B55^ holoenzymes, thus preventing dephosphorylation of substrates critical for cell survival. This effect might be informative in terms of PP2A function and, as E4orf4 kills human cancer cells preferentially, of potential importance for the development of new cancer therapies related to regulation of PP2A. Nevertheless, we consider that this toxicity represents an artifact of overexpression relative to its function during adenovirus replication.

We believe that the function of the E4orf4 protein for the virus is largely to deliver critical substrates to B/B55-containing PP2A holoenzymes. To this end we examined one substrate the dephosphorylation of which was known to be affected by E4orf4, ASF/SF2/SRSF1, an SR protein that mediates alternative splicing of late viral mRNAs [58]. Studies involving E4orf4 mutants suggested that an interaction between E4orf4, ASF/SF2/SRSF1, and an active PP2A complex was required for E4orf4-induced SR protein dephosphorylation [59]. Confirming these early results, we showed that E4orf4 targets ASF/SF2/SRSF1 to PP2A^B55α^ (Figure 8B and 8D). Given these findings we believe that perhaps many additional targets that optimize adenovirus replication remain to be identified.

## Materials and Methods

### Cell lines and yeast strains

Human H1299 (p53^−/−^) cells (ATCC CRL-5803) were cultured in Dulbecco's Modified Eagle's Medium with 4.5 g/L glucose, L-glutamine and sodium pyruvate (Wisent) supplemented with 10% fetal calf serum (Wisent). Yeast strain YS95 [41], from a W303 background (*MAT*α *ade2-1 ura3-1 his3-11 leu2-3,112 cdc55::TRP1*), was used and yeast cell growth assays were performed as previously described [14].

### Plasmids and viruses

DNA plasmids pcDNA3-HA-E4orf4 and pcDNA3-FLAG-B55α for mammalian cell expression have previously been described [7]. Throughout, we have used cDNAs expressing rat B55α, which is identical in amino acid sequence to human B55α except for the conservative change of Ile310 to Val. B55α point mutants with amino acid substitutions were generated by site-directed PCR mutagenesis, with conditions as previously described [49]. The pEGFP-SF2 plasmid was obtained through Addgene (plasmid 17990).

Cdc55 point mutants with amino acid substitutions were generated in a similar fashion, also using site-directed PCR mutagenesis. Plasmid DNA used was p415*CDC55pr*-*FLAG-CDC55* as template. This plasmid was created with the p415*GAL1* vector (ATCC), where the *GAL1* promoter was removed by restriction enzyme digestion with *SacI*/*BamHI* and replaced by the endogenous *CDC55* promoter (500 bp) using primers Fwd – 5′-GAATTCGAGCTCTGCTGATGCCGCCAAGAAG-3′, and Rev – 5′-CGGGATCCCATTGTGCGCTATATTATATTTC-3′, subcloned from the pRS425*CDC55* plasmid (a gift from Howard Bussey, McGill University). *FLAG-CDC55* was subcloned into this p415*CDC55pr* vector through *HindIII*/*XhoI* restriction enzyme digests of pcDNA3-*FLAG-CDC55*. HA-E4orf4 was subcloned into the p426*GAL1* or pYES2 expression vector as previously described [14].

Construction of FLAG-E4orf4 and E4orf4- viruses was described previously [57]. It is derived from the wild type virus H5pg4100 which is partially deleted for the E3 region and adds a FLAG tag in frame at the carboxyl terminus of the E4orf4 protein.

### Mammalian cell DNA transfections and yeast cell transformations

H1299 cells plated onto 100 mm diameter dishes and transfected with plasmid DNAs using Lipofectamine 2000 (Invitrogen), as specified by the manufacturer. Three µl of Lipofectamine 2000 reagent was used per 1 µg of DNA transfected. For all experiments, 3 µg of pcDNA3-FLAG-B55α species (wild type or mutant) and 3 µg of pcDNA3-HA-E4orf4 (wild type or mutant species) were transfected.

Aforementioned yeast expression plasmids were transformed into YS95 as previously described [14]. Briefly, yeast transformations were performed using the one-step method [60]. Transformants were selected on synthetic complete media containing 2% glucose and the appropriate auxotrophic supplements.

### Cell lysis, co-immunoprecipitations and western blotting

H1299 cells were harvested 24 h post-transfection and lysed for 30 min on ice with lysis buffer (20 mM Tris-HCl, pH 7.5, 150 mM NaCl, 2 mM EDTA, 1% Triton X-100, 5% glycerol) supplemented with 2 mM DTT, 4 mM NaF, 2 mM NaPP, 500 µM Na_3_VO_4_, 200 µg/ml PMSF, 2 µg/ml aprotinin, 5 µg/ml leupeptin. 500 µg-1 mg of protein extract was used for co-immunoprecipitations. Antibodies used in co-immunoprecipitations were as follows: mouse monoclonal anti-HA (HA.11, Covance), mouse monoclonal anti-FLAG (M2, Sigma-Aldrich), mouse monoclonal anti-GFP (JL-8, Living Colors), and mouse monoclonal anti-SF2/ASF (Zymed, 32-4500). Protein extracts were pre-cleared with a 50% Protein G/50% Protein A agarose slurry (Millipore). Incubation of lysates with appropriate antibodies was followed by incubation with Protein A/G slurry. Immunoprecipitates were washed five times with lysis buffer, heated in sample buffer for 5 min, followed by SDS-PAGE, transfer to PVDF membranes (Millipore) and western blotting with appropriate antibodies. Whole cell extracts (WCE) were prepared using 20–50 µg of total protein. Antibodies used for western blotting included: rabbit polyclonal anti-FLAG (Sigma-Aldrich), rabbit polyclonal anti-HA (Sigma-Aldrich), anti-GFP mouse monoclonal (JL-8, Living Colors), rabbit polyclonal anti-p107 (C-18, Santa Cruz), rabbit polyclonal anti-PP2A A (07-250, Millipore), and mouse monoclonal PP2A B55α (2G9, Upstate). Membranes were incubated with secondary antibody linked to horse radish peroxidase (Jackson ImmunoResearch) followed by ECL detection (PerkinElmer).

Yeast cells were cultured as previously described [14]. Briefly, yeast were grown in 2% glucose-containing medium overnight, transferred to 2% raffinose for 2 h and resuspended in fresh medium containing 2% raffinose and 2% galactose for 6 h to induce E4orf4 expression. Cells were then harvested and lysates were prepared as previously described [14]. For co-immunoprecipitations, 1 mg of lysate was pre-cleared with a 50% Protein A/50% Protein G Agarose slurry (Millipore) and then incubated with anti-HA or anti-FLAG antibodies before being washed five times with lysis buffer, heated in sample buffer for 5 min, followed by SDS-PAGE, PVDF transfer and western blotting with appropriate antibodies. WCE were prepared with 20–50 µg of total protein extract. Immunoblotting was performed with the indicated antibodies. Anti-HA (HA.11, Covance), anti-FLAG (M2, Sigma-Aldrich), and anti-Tpd3 (a gift from James R. Broach, Princeton) antibodies were all used.

### Rendering of Cdc55 and B55α structures

The PP2A^B55α^ structure was obtained from the Protein Data Bank (DOI: 10.2210/pdb3dw8/pdb). The predicted structure of Cdc55 was generated using ModWeb Version SVN.r1368M, a server for protein structure modeling based on Modeller software [61]. The structure of B55α, PP2A^B55α^ and the predicted structure of Cdc55 were viewed and modified using the PyMol Molecular Graphics System, Version 1.20 (DeLano Scientific, Schrödinger, LLC).

## Supporting Information

Figure S1 **Cell growth assays for** ***north*** **Cdc55 mutants.** Cell growth assays were conducted in the presence of empty vector or E4orf4-expressing plasmid DNA in a *cdc55Δ* background yeast strain (YS95) exogenously expressing either empty vector, wild type (WT) Cdc55, or Cdc55 *north* mutants (D342K, E345K, D347K, D342K/E345K/D347K) under its normal promoter. Cell growth assays were performed on three different types of growth conditions: 2% glucose medium as a control where E4orf4 is not expressed (**A**); 2% glucose medium containing 100 nM rapamycin to test the PP2A holoenzyme-forming capability of the Cdc55 species (**B**); and 2% raffinose/galactose medium to induce E4orf4 expression (**C**). Results are summarized in Table 3.
(EPS)

Figure S2 **Cell growth assays for** ***south*** **Cdc55 mutants.** Cell growth assays similar to those in Supplementary Figure S1 were conducted in the presence of empty vector or E4orf4-expressing plasmid DNA in a *cdc55Δ* background yeast strain (YS95) exogenously expressing either empty vector, wild type (WT) Cdc55, or the different Cdc55 *south* mutants (E82K, E84K, E85K, K86E, E82K/E84K/E85K/K86E) under its normal promoter. Cell growth assays were performed on three different types of growth conditions: 2% glucose medium as a control where E4orf4 is not expressed (**A**); 2% glucose medium containing 100 nM Rapamycin to test the PP2A holoenzyme-forming capability of the Cdc55 species (**B**); and 2% raffinose/galactose medium to induce E4orf4 expression (**C**). Results are summarized in Table 3.
(EPS)

## References

[1] Marcellus RC, Teodoro JG, Wu T, Brough DE, Ketner G, et al (1996) Adenovirus type 5 early region 4 is responsible for E1A-induced p53-independent apoptosis. Journal Of Virology 70: 6207–6215. 8709247 10.1128/jvi.70.9.6207-6215.1996 PMC190645

[2] Lavoie JN, Nguyen M, Marcellus RC, Branton PE, Shore GC (1998) E4orf4, a novel adenovirus death factor that induces p53-independent apoptosis by a pathway that is not inhibited by zVAD-fmk. Journal Of Cell Biology 140: 637–645. 9456323 10.1083/jcb.140.3.637 PMC2140159

[3] Marcellus RC, Lavoie JN, Boivin D, Shore GC, Ketner G, et al (1998) The early region 4 orf4 protein of human adenovirus type 5 induces p53-independent cell death by apoptosis. Journal Of Virology 72: 7144–7153. 9696808 10.1128/jvi.72.9.7144-7153.1998 PMC109936

[4] Shtrichman R, Kleinberger T (1998) Adenovirus type 5 E4 open reading frame 4 protein induces apoptosis in transformed cells. Journal Of Virology 72: 2975–2982. 9525619 10.1128/jvi.72.4.2975-2982.1998 PMC109744

[5] Shtrichman R, Sharf R, Barr H, Dobner T, Kleinberger T (1999) Induction of apoptosis by adenovirus E4orf4 protein is specific to transformed cells and requires an interaction with protein phosphatase 2A. Proceedings Of The National Academy Of Sciences Of The United States Of America 96: 10080–10085. 10468565 10.1073/pnas.96.18.10080 PMC17845

[6] Lavoie JN, Champagne C, Gingras MC, Robert A (2000) Adenovirus E4 open reading frame 4-induced apoptosis involves dysregulation of Src family kinases. Journal of Cell Biology 150: 1037–1056. 10973994 10.1083/jcb.150.5.1037 PMC2175248

[7] Marcellus RC, Chan H, Paquette D, Thirlwell S, Boivin D, et al (2000) Induction of p53-independent apoptosis by the adenovirus E4orf4 protein requires binding to the Balpha subunit of protein phosphatase 2A. J Virol 74: 7869–7877. 10933694 10.1128/jvi.74.17.7869-7877.2000 PMC112317

[8] Shtrichman R, Sharf R, Kleinberger T (2000) Adenovirus E4orf4 protein interacts with both Balpha and B′ subunits of protein phosphatase 2A, but E4orf4-induced apoptosis is mediated only by the interaction with Balpha. Oncogene 19: 3757–3765. 10949930 10.1038/sj.onc.1203705

[9] Kornitzer D, Sharf R, Kleinberger T (2001) Adenovirus E4orf4 protein induces PP2A-dependent growth arrest in Saccharomyces cerevisiae and interacts with the anaphase-promoting complex/cyclosome. J Cell Biol 154: 331–344. 11470822 10.1083/jcb.200104104 PMC2150760

[10] Livne A, Shtrichman R, Kleinberger T (2001) Caspase activation by adenovirus e4orf4 protein is cell line specific and Is mediated by the death receptor pathway. Journal of Virology 75: 789–798. 11134292 10.1128/JVI.75.2.789-798.2001 PMC113975

[11] Roopchand DE, Lee JM, Shahinian S, Paquette D, Bussey H, et al (2001) Toxicity of human adenovirus E4orf4 protein in Saccharomyces cerevisiae results from interactions with the Cdc55 regulatory B subunit of PP2A. Oncogene 20: 5279–5290. 11536041 10.1038/sj.onc.1204693

[12] Li S, Brignole C, Marcellus R, Thirlwell S, Binda O, et al (2009) The adenovirus E4orf4 protein induces G2/M arrest and cell death by blocking protein phosphatase 2A activity regulated by the B55 subunit. J Virol 83: 8340–8352. 19535438 10.1128/JVI.00711-09 PMC2738146

[13] Li S, Szymborski A, Miron MJ, Marcellus R, Binda O, et al (2009) The adenovirus E4orf4 protein induces growth arrest and mitotic catastrophe in H1299 human lung carcinoma cells. Oncogene 28: 390–400. 18955965 10.1038/onc.2008.393

[14] Mui MZ, Roopchand DE, Gentry MS, Hallberg RL, Vogel J, et al (2010) Adenovirus protein E4orf4 induces premature APCCdc20 activation in Saccharomyces cerevisiae by a protein phosphatase 2A-dependent mechanism. J Virol 84: 4798–4809. 20164229 10.1128/JVI.02434-09 PMC2863776

[15] Li Y, Wei H, Hsieh TC, Pallas DC (2008) Cdc55p-mediated E4orf4 growth inhibition in Saccharomyces cerevisiae is mediated only in part via the catalytic subunit of protein phosphatase 2A. J Virol 82: 3612–3623. 18216111 10.1128/JVI.02435-07 PMC2268493

[16] Robert A, Miron MJ, Champagne C, Gingras MC, Branton PE, et al (2002) Distinct cell death pathways triggered by the adenovirus early region 4 ORF 4 protein. J Cell Biol 158: 519–528. 12163473 10.1083/jcb.200201106 PMC2173819

[17] Pechkovsky A, Lahav M, Bitman E, Salzberg A, Kleinberger T (2013) E4orf4 induces PP2A- and Src-dependent cell death in Drosophila melanogaster and at the same time inhibits classic apoptosis pathways. Proceedings Of The National Academy Of Sciences Of The United States Of America 110: E1724–1733. 23613593 10.1073/pnas.1220282110 PMC3651459

[18] Cabon L, Sriskandarajah N, Mui MZ, Teodoro JG, Blanchette P, et al (2013) Adenovirus E4orf4 Protein-Induced Death of p53−/− H1299 Human Cancer Cells Follows a G1 Arrest of Both Tetraploid and Diploid Cells Due to a Failure to Initiate DNA Synthesis. Journal Of Virology 01242–13. 10.1128/JVI.01242-13 PMC3838244 24067978

[19] Brestovitsky A, Sharf R, Mittelman K, Kleinberger T (2011) The adenovirus E4orf4 protein targets PP2A to the ACF chromatin-remodeling factor and induces cell death through regulation of SNF2h-containing complexes. Nucleic acids research 39: 6414–6427. 21546548 10.1093/nar/gkr231 PMC3159439

[20] Kleinberger T, Shenk T (1993) Adenovirus E4orf4 protein binds to protein phosphatase 2A, and the complex down regulates E1A-enhanced junB transcription. Journal Of Virology 67: 7556–7560. 8230475 10.1128/jvi.67.12.7556-7560.1993 PMC238222

[21] Koren R, Rainis L, Kleinberger T (2004) The scaffolding A/Tpd3 subunit and high phosphatase activity are dispensable for Cdc55 function in the Saccharomyces cerevisiae spindle checkpoint and in cytokinesis. J Biol Chem 279: 48598–48606. 15347656 10.1074/jbc.M409359200

[22] Afifi R, Sharf R, Shtrichman R, Kleinberger T (2001) Selection of apoptosis-deficient adenovirus E4orf4 mutants in Saccharomyces cerevisiae. Journal of Virology 75: 4444–4447. 11287598 10.1128/JVI.75.9.4444-4447.2001 PMC114194

[23] Horowitz B, Sharf R, Avital-Shacham M, Pechkovsky A, Kleinberger T (2013) Structure- and modeling-based identification of the adenovirus E4orf4 binding site in the protein phosphatase 2A (PP2A) B55alpha subunit. journal of biological chemistry 288: 13718–13727. 23530045 10.1074/jbc.M112.343756 PMC3650409

[24] Zhang Z, Mui MZ, Chan F, Roopchand DE, Marcellus RC, et al (2011) Genetic analysis of B55alpha/Cdc55 protein phosphatase 2A subunits: association with the adenovirus E4orf4 protein. J Virol 85: 286–295. 21047956 10.1128/JVI.01381-10 PMC3014202

[25] Mumby MC, Walter G (1993) Protein serine/threonine phosphatases: structure, regulation, and functions in cell growth. physiological reviews 73: 673–699. 8415923 10.1152/physrev.1993.73.4.673

[26] Stark MJR (1996) Yeast protein serine threonine phosphatases: multiple roles and diverse regulation. Yeast 12: 1647–1675. 9123967 10.1002/(SICI)1097-0061(199612)12:16%3C1647::AID-YEA71%3E3.0.CO;2-Q

[27] Goldberg Y (1999) Protein Phosphatase 2A: Who Shall Regulate the Regulator. Biochemical Pharmacology 57: 321–328. 9933020 10.1016/s0006-2952(98)00245-7

[28] Lechward K, Zolnierowicz S, Hemmings BA (1999) Eukaryotic translation termination factor 1 associates with protein phosphatase 2A and targets it to ribosomes. biochemistry (mosc) 64: 1373–1381. 10648961

[29] Janssens V, Goris J (2001) Protein phosphatase 2A: a highly regulated family of serine/threonine phosphatases implicated in cell growth and signalling. Biochemical Journal 353: 417–439. 11171037 10.1042/0264-6021:3530417 PMC1221586

[30] Kurimchak A, Grana X (2012) PP2A holoenzymes negatively and positively regulate cell cycle progression by dephosphorylating pocket proteins and multiple CDK substrates. Gene 499: 1–7. 22387205 10.1016/j.gene.2012.02.015 PMC7527233

[31] Sablina AA, Hector M, Colpaert N, Hahn WC (2010) Identification of PP2A complexes and pathways involved in cell transformation. Cancer Res 70: 10474–10484. 21159657 10.1158/0008-5472.CAN-10-2855 PMC3056544

[32] Westermarck J, Hahn WC (2008) Multiple pathways regulated by the tumor suppressor PP2A in transformation. Trends Mol Med 14: 152–160. 18329957 10.1016/j.molmed.2008.02.001

[33] Ferrigno P, Langan TA, Cohen P (1993) Protein phosphatase 2A1 is the major enzyme in vertebrate cell extracts that dephosphorylates several physiological substrates for cyclin-dependent protein kinases. molecular biology of the cell 4: 669–677. 8400454 10.1091/mbc.4.7.669 PMC300977

[34] Kamibayashi C, Estes R, Lickteig RL, Yang SI, Craft C, et al (1994) Comparison of heterotrimeric protein phosphatase 2A containing different B subunits. journal of biological chemistry 269: 20139–20148. 8051102

[35] Janssens V, Longin S, Goris J (2008) PP2A holoenzyme assembly: in cauda venenum (the sting is in the tail). Trends Biochem Sci 33: 113–121. 18291659 10.1016/j.tibs.2007.12.004

[36] Sents W, Ivanova E, Lambrecht C, Haesen D, Janssens V (2013) The biogenesis of active protein phosphatase 2A holoenzymes: a tightly regulated process creating phosphatase specificity. FEBS J 280: 644–661. 22443683 10.1111/j.1742-4658.2012.08579.x

[37] Virshup DM, Shenolikar S (2009) From promiscuity to precision: protein phosphatases get a makeover. Mol Cell 33: 537–545. 19285938 10.1016/j.molcel.2009.02.015

[38] Sneddon AA, Cohen PT, Stark MJ (1990) Saccharomyces cerevisiae protein phosphatase 2A performs an essential cellular function and is encoded by two genes. embo journal 9: 4339–4346. 2176150 10.1002/j.1460-2075.1990.tb07883.x PMC552220

[39] Ronne H, Carlberg M, Hu GZ, Nehlin JO (1991) Protein phosphatase 2A in Saccharomyces cerevisiae: effects on cell growth and bud morphogenesis. Molecular And Cellular Biology 11: 4876–4884. 1656215 10.1128/mcb.11.10.4876-4884.1991 PMC361456

[40] van Zyl W, Huang W, Sneddon AA, Stark M, Camier S, et al (1992) Inactivation of the protein phosphatase 2A regulatory subunit A results in morphological and transcriptional defects in Saccharomyces cerevisiae. Molecular And Cellular Biology 12: 4946–4959. 1328868 10.1128/mcb.12.11.4946-4959.1992 PMC360427

[41] Shu Y, Yang H, Hallberg E, Hallberg R (1997) Molecular genetic analysis of Rts1p, a B′ regulatory subunit of Saccharomyces cerevisiae protein phosphatase 2A. Molecular And Cellular Biology 17: 3242–3253. 9154823 10.1128/mcb.17.6.3242 PMC232177

[42] Healy AM, Zolnierowicz S, Stapleton AE, Goebl M, DePaoli-Roach AA, et al (1991) CDC55, a Saccharomyces cerevisiae gene involved in cellular morphogenesis: identification, characterization, and homology to the B subunit of mammalian type 2A protein phosphatase. Molecular And Cellular Biology 11: 5767–5780. 1656238 10.1128/mcb.11.11.5767-5780.1991 PMC361948

[43] Arroyo JD, Hahn WC (2005) Involvement of PP2A in viral and cellular transformation. Oncogene 24: 7746–7755. 16299534 10.1038/sj.onc.1209038

[44] Pallas DC, Cherington V, Morgan W, DeAnda J, Kaplan D, et al (1988) Cellular proteins that associate with the middle and small T antigens of polyomavirus. Journal Of Virology 62: 3934–3940. 2845116 10.1128/jvi.62.11.3934-3940.1988 PMC253819

[45] Pallas DC, Shahrik LK, Martin BL, Jaspers S, Miller TB, et al (1990) Polyoma small and middle T antigens and SV40 small t antigen form stable complexes with protein phosphatase 2A. Cell 60: 167–176. 2153055 10.1016/0092-8674(90)90726-u

[46] Walter G, Ruediger R, Slaughter C, Mumby M (1990) Association of protein phosphatase 2A with polyoma virus medium tumor antigen. Proceedings Of The National Academy Of Sciences Of The United States Of America 87: 2521–2525. 2157202 10.1073/pnas.87.7.2521 PMC53721

[47] Yang SI, Lickteig RL, Estes R, Rundell K, Walter G, et al (1991) Control of protein phosphatase 2A by simian virus 40 small-t antigen. molecular and cellular biology 11: 1988–1995. 1706474 10.1128/mcb.11.4.1988-1995.1991 PMC359884

[48] Ruediger R, Roeckel D, Fait J, Bergqvist A, Magnusson G, et al (1992) Identification of binding sites on the regulatory A subunit of protein phosphatase 2A for the catalytic C subunit and for tumor antigens of simian virus 40 and polyomavirus. molecular and cellular biology 12: 4872–4882. 1328865 10.1128/mcb.12.11.4872-4882.1992 PMC360420

[49] Miron MJ, Gallouzi IE, Lavoie JN, Branton PE (2004) Nuclear localization of the adenovirus E4orf4 protein is mediated through an arginine-rich motif and correlates with cell death. Oncogene 23: 7458–7468. 15334069 10.1038/sj.onc.1207919

[50] Xu Y, Chen Y, Zhang P, Jeffrey PD, Shi Y (2008) Structure of a protein phosphatase 2A holoenzyme: insights into B55-mediated Tau dephosphorylation. Mol Cell 31: 873–885. 18922469 10.1016/j.molcel.2008.08.006 PMC2818795

[51] Xing Y, Xu Y, Chen Y, Jeffrey PD, Chao Y, et al (2006) Structure of protein phosphatase 2A core enzyme bound to tumor-inducing toxins. Cell 127: 341–353. 17055435 10.1016/j.cell.2006.09.025

[52] O'Shea C, Klupsch K, Choi S, Bagus B, Soria C, et al (2005) Adenoviral proteins mimic nutrient/growth signals to activate the mTOR pathway for viral replication. The EMBO journal 24: 1211–1221. 15775987 10.1038/sj.emboj.7600597 PMC556401

[53] Kleinberger T (2004) Induction of transformed cell-specific apoptosis by the adenovirus E4orf4 protein. Prog Mol Subcell Biol 36: 245–267. 15171615 10.1007/978-3-540-74264-7_12

[54] Jayadeva G, Kurimchak A, Garriga J, Sotillo E, Davis AJ, et al (2010) B55alpha PP2A holoenzymes modulate the phosphorylation status of the retinoblastoma-related protein p107 and its activation. J Biol Chem 285: 29863–29873. 20663872 10.1074/jbc.M110.162354 PMC2943288

[55] Kurimchak A, Haines DS, Garriga J, Wu S, De Luca F, et al (2013) Activation of p107 by FGF, which is essential for chondrocyte cell cycle exit, is mediated by the PP2A/B55alpha holoenzyme. molecular and cellular biology doi:10.1128/MCB.00082-13 10.1128/MCB.00082-13 PMC3753905 23775125

[56] Kolupaeva V, Daempfling L, Basilico C (2013) The B55 alpha regulatory subunit of Protein Phosphatase 2A mediates FGF-induced p107 dephosphorylation and growth arrest in chondrocytes. molecular and cellular biology doi:10.1128/MCB.01730-12 10.1128/MCB.01730-12 PMC3719682 23716589

[57] Miron MJ, Blanchette P, Groitl P, Dallaire F, Teodoro JG, et al (2009) Localization and importance of the adenovirus E4orf4 protein during lytic infection. Journal Of Virology 83: 1689–1699. 19073741 10.1128/JVI.01703-08 PMC2643762

[58] Kanopka A, Mühlemann O, Petersen-Mahrt S, Estmer C, Ohrmalm C, et al (1998) Regulation of adenovirus alternative RNA splicing by dephosphorylation of SR proteins. Nature 393: 185–187. 9603524 10.1038/30277

[59] Estmer Nilsson C, Petersen-Mahrt S, Durot C, Shtrichman R, Krainer AR, et al (2001) The adenovirus E4-ORF4 splicing enhancer protein interacts with a subset of phosphorylated SR proteins. The EMBO journal 20: 864–871. 11179230 10.1093/emboj/20.4.864 PMC145406

[60] Chen DC, Yang BC, Kuo TT (1992) One-step transformation of yeast in stationary phase. Current Genetics 21: 83–84. 1735128 10.1007/BF00318659

[61] Sali A, Blundell TL (1993) Comparative protein modelling by satisfaction of spatial restraints. J Mol Biol 234: 779–815. 8254673 10.1006/jmbi.1993.1626

